# Associations between testicular development and fetal size in the pig

**DOI:** 10.1186/s40104-022-00678-3

**Published:** 2022-03-15

**Authors:** Claire Stenhouse, Yennifer Cortes-Araya, F. Xavier Donadeu, Cheryl J. Ashworth

**Affiliations:** 1 Functional Genetics and Development Division, The Roslin Institute and Royal (Dick) School of Veterinary Studies, University of Edinburgh, Midlothian, UK; 2 Department of Animal Science, Texas A&M University, 440 Kleberg Center, College Station, TX 77843-2471 USA

**Keywords:** Fetal growth, Intrauterine growth restriction (IUGR), Porcine, Pregnancy, Testes

## Abstract

**Background:**

Impaired reproductive performance is the largest contributing factor for the removal of boars from commercial systems. Intrauterine growth restricted piglets represent 25% of the total number of piglets born and have impaired reproductive performance. This study aimed to improve the understanding of temporal changes in testicular gene expression during testes development in fetuses of different size. The lightest and closest to mean litter weight (CTMLW) male Large White × Landrace littermates were collected at gestational days (GD) 45, 60 and 90 (*n* = 5–6 litters/GD).

**Results:**

Testes weight and testes weight as a percentage of fetal weight were not associated with fetal size at GD60 or 90. Fetal plasma testosterone was not associated with fetal size at GD90. There was no association between fetal size and seminiferous tubule area and number, number of germ or Sertoli cells per tubule. The lightest fetuses tended to have wider seminiferous tubules compared to the CTMLW fetuses at GD90 (*P* = 0.077). The testicular expression of *KI67* (*P* ≤ 0.01) and *BAX:BCL2* ratio (*P* = 0.058) mRNAs decreased as gestation progressed. Greater *SPP1* mRNA expression was observed at GD60 when compared with GD45 and 90 (*P* ≤ 0.05). Lower expression of *DMRT1* and *SPP1* (*P* < 0.01) mRNAs was observed in testes associated with the lightest fetuses compared to the CTMLW fetuses at GD90.

**Conclusions:**

These findings provide novel insights into the expression profiles of genes associated with testicular development and function. Further, these data suggest that programming of reproductive potential in IUGR boars occurs late in gestation, providing a platform for further mechanistic investigation.

**Supplementary Information:**

The online version contains supplementary material available at 10.1186/s40104-022-00678-3.

## Background

Artificial insemination is extensively utilised to improve herd genetics and fertility, which has proved to be a successful tactic to increase the profitability of the pig industry [1, 2]. To maximise the profitability of a commercial pig system, it is essential that the boars utilised as semen donors have their fertility monitored. Impaired reproductive performance is the largest contributing factor for the removal of boars from commercial systems therefore, to maximise productivity, it is essential that boars with poor reproductive performance are identified as early as possible. Significant developmental changes must occur during development of the mammalian testes [3, 4], and perturbations in these processes prenatally can have significant consequences for fertility postnatally [5, 6]. Therefore, improving the understanding of the mechanisms regulating fetal testes development is critical to improve the understanding of the regulation of postnatal boar fertility, and is vital to improve the success of the pig industry.

It has been proposed that birth weight could be an easily applicable criterion for the selection of boars with the greatest reproductive potential. An association between birth weight and testicular weight and/or volume has been reported in both neonatal and adult boars [7–9]. Auler et al. [7] demonstrated that heavier male littermates have greater numbers of somatic and germ cells compared to their lightest male littermates on postnatal day 8, despite no alterations in circulatory testosterone or testicular 17α-hydroxylase expression. Further, despite a lack of differences in circulatory testosterone or testicular 17α-hydroxylase expression at 8 months of age, the lighter birthweight males had decreased numbers of pachytene spermatocytes and round spermatids, accompanied by decreased total numbers of elongated spermatids and daily sperm production.

To increase pig production, genetic selection for favourable traits such as litter size and ovulation rate has been utilised to increase the number of piglets weaned per sow per year [10]. Whilst this strategy has increased the number of live born piglets, there is substantial evidence that uterine capacity has not increased as anticipated, which hinders both litter size and piglet weight due to inadequate placental growth and efficiency [11, 12]. This has led to substantial within-litter variation in piglet weight and a high prevalence of low birthweight piglets; with approximately 25% of the total number of piglets born considered intrauterine growth restricted (IUGR) [13]. It has been suggested that IUGR in the pig may be programmed from an early stage of gestation, with significant within-litter variation in fetal size observed from as early as gestational day 30–35 [14–18]. IUGR in the pig severely impacts neonatal and adult development, with piglets exhibiting increased morbidity and mortality [12]. In addition, this phenotype has been suggested to be associated with impaired reproductive performance in boars, although this concept remains under-investigated. IUGR boars have been reported to have impaired testicular development, with decreased numbers of germ cells, testicular volume, semen volume, and fewer spermatozoa per ejaculate postnatally when compared to normally-grown piglets [8, 9]. Considering the high prevalence and severity of the phenotype, it is essential to improve the understanding of the mechanisms associated with impaired testis development in the lightest fetuses compared to their normal-sized littermates [19].

Whilst Pontelo et al. [20] recently described temporal changes in testicular size and histological structure during porcine fetal development, the expression profiles of genes associated with testicular development and function remain poorly understood. Significant changes in the structure of the testes occur during gestation to prepare for their essential role in male fertility later in life. In this study, the temporal expression profile of mRNAs with central roles in apoptosis, proliferation, and the testicular extracellular matrix was determined. Despite the appreciation that angiogenesis is essential for organ development and growth, and the essential role of angiogenesis in the postnatal testes, little is known regarding vascularisation of the fetal testes. Considering this, several of the candidate genes investigated in this study have central roles in the regulation of angiogenesis. Given the high prevalence of growth-restricted piglets, and the postnatal reproductive phenotype associated with IUGR, it is essential that we improve our understanding of the mechanisms governing fetal testicular development to improve the reproductive efficiency of the pig industry.

This study aimed to improve the understanding of temporal changes in testicular gene expression during fetal testes development. Further, it was hypothesised that the developmental trajectory of testicular development deviates between the lightest and normal sized fetuses.

## Methods

All procedures were performed with approval from The Roslin Institute (University of Edinburgh) Animal Welfare and Ethical Review Board and in accordance with the U.K. Animals (Scientific Procedures) Act, 1986.

### Experimental animals and sample collection

Large White × Landrace gilts (age 11–14 months) were observed daily for signs of oestrus and were housed in groups of 6–8 animals per pen. Oestrous cyclicity and ovarian function were controlled in accordance with routine normal practice at The Roslin Institute Large Animal Unit (Roslin, Midlothian, Scotland). All gilts were inseminated twice daily for the duration of oestrus with semen from one of four Large White sires. The sires used were equally distributed between gilts at the different gestational days (GD) of interest to minimise any effect of sire. The first day of insemination was assigned as GD0. Gilts were humanely killed with sodium pentobarbitone 20% w/v (Henry Schein Animal Health, Dumfries, Dumfries and Galloway, U.K.) at a dose of 0.4 mL/kg by intravenous injection via a cannula inserted in the ear vein at GD45, 60 and 90 (*n* = 5–6 gilts/GD).

Following confirmation of death, mid-ventral incision revealed the reproductive tract which was lifted from the body cavity and placed in a dissecting tray. Both uterine horns were dissected, from the ovary towards the cervix. All fetuses were weighed to identify the lightest and closest to mean litter weight (CTMLW) fetuses, with sex determined morphologically and confirmed by PCR for the SRY region of the Y chromosome as described previously [21].

For all developmental stages, the testes were dissected for analyses. At GD45, the testes were not weighed, but at GD60 and 90 the testes were weighed. One testis from each fetus was snap frozen in liquid nitrogen and stored at – 80 °C until required, and the other testis was fixed in Bouin’s for histological analysis (GD60 and 90 only). In addition, cardiac puncture was performed using an EDTA coated syringe to collect blood samples from the lightest and CTMLW male fetuses at GD90 (*n* = 5 litters). Plasma was obtained from fetal blood samples by centrifugation and samples were stored at − 20 °C until required.

### Plasma testosterone quantification

Testosterone concentrations were determined in fetal plasma samples from the lightest and CTMLW male fetuses at GD90 (*n* = 5 CTMLW fetuses; 5 lightest fetuses) using an ELISA kit that has been validated for use with porcine samples (Abscitech, College Park, MD, USA; EK0379), as per the manufacturer’s instructions. The detection range of the assay was 0.1–20 ng/mL, with a sensitivity of 0.05 ng/mL.

### Histological analysis

Testes samples from GD60 (*n* = 5 CTMLW fetuses; 5 lightest fetuses) and 90 (*n* = 6 CTMLW fetuses; 6 lightest fetuses) were fixed with Bouin’s overnight at room temperature and changed daily for approximately 1 week in 70% ethanol (Genta Medical, York, U.K.). Testes were processed by passing through graded ethanol (70%, 95%, and 99%; Genta Medical) and xylene (Genta Medical). The samples were then embedded in paraffin wax (Fisher Scientific, Loughborough, Leicestershire, U.K.) and 5 μm sections were cut and placed on polysine microscope slides (Fisher Scientific).

Following dewaxing and heat-induced epitope retrieval in 0.01 mol/L sodium citrate (Vector Laboratories), endogenous peroxidase activity was blocked by incubating slides with 0.3% hydrogen peroxide (Sigma Aldrich) in methanol. Non-specific binding sites were blocked by incubation with normal goat serum (Vectastain Elite ABC kit; Vector Laboratories, Orton Southgate, Peterborough, U.K.). Sections were incubated with a primary antibody for GATA binding protein 4 (GATA4) (sc-9053; Santa Cruz, Heidelberg, Germany) at a 1:200 dilution, or with rabbit immunoglobulin G (RIgG) (Vector Laboratories) (equivalent total protein concentration) as a negative control (Additional file 1: Supplementary Fig. 1). The slides were incubated in a humidified chamber at 4 °C overnight, washed in phosphate buffered saline (PBS), and incubated for 30 min at room temperature with a biotinylated anti-rabbit IgG secondary antibody (Vectastain Elite ABC kit; Vector Laboratories) at a dilution of 1:200 in PBS containing 1.5% normal goat serum. Sections were incubated with Vectastain Elite ABC reagent (Vectastain Elite ABC kit; Vector Laboratories) for 30 min, before incubation with the Novared peroxidase substrate (Vector Laboratories) for 5 min. Sections were then counterstained with haematoxylin and dehydrated in a graded series of ethanol and xylene (70%, 95%, and 99% ethanol; 99% ethanol 1:1 with xylene, and absolute xylene; Genta Medical). The sections were imaged using the NanoZoomer slide scanner (Hamamatsu, Shizuoka, Japan).

### Image analysis

All image analyses were performed using ImageJ. Six images were taken at × 20 magnification for each section. For each testis, 2 sections were analysed which were a minimum of 5 serial sections apart from one another. The number of tubules were quantified and expressed as number per 0.1 mm^2^. For each tubule, the mean diameter was calculated by taking the average of 3 measurements. The total area for each tubule, and the number of germ cells and Sertoli cells per tubule were quantified.

### Total RNA extraction and cDNA synthesis

RNA was extracted from the whole snap-frozen testis sample from each fetus as described previously [22–24]. The RNA was quantified, and the quality assessed spectrophotometrically using a Nanodrop ND-1000 (Labtech International Ltd., Heathfield, East Sussex, U.K.) and electrophoretically using a Tapestation 2200 (Agilent Technologies, Edinburgh, U.K.) (RNA Integrity Number Equivalent (RINe); 9.048 ± 0.092). Extracted RNA was stored at – 80 °C until required.

Complementary DNA (cDNA) was prepared from 0.3 μg of RNA with SuperScript III reverse transcriptase (Life Technologies, Paisley, Renfrewshire, U.K.) following the manufacturer’s instructions. Each reaction contained 250 ng random primers (Promega, Chilworth, Hampshire, U.K.) and 40 units RNaseIn (Promega). Negative controls without reverse transcriptase were included to check for genomic contamination and all cDNA was stored at – 20 °C until required.

### Relative expression of candidate genes in testicular samples

The expression of candidate mRNAs was quantified  by qPCR in testes samples associated with the lightest and CTMLW male fetuses at GD45, 60 and 90. Quantitative PCR was performed on a Stratagene MX3000 instrument using SensiFAST® SYBR Lo-ROX (Bioline, London, U.K.) and cDNA from fetal testes at GD45, 60 and 90. All qPCRs were carried out at an annealing temperature of 60 °C and dissociation curves consisting of single peaks were generated. Candidate mRNAs were quantified: BCL-2-associated X protein (*BAX*), b-cell lymphoma 2 (*BCL2*), platelet endothelial cell adhesion molecule (*CD31*), doublesex and mab-3 related transcription factor 1 (*DMRT1*), GATA binding protein 4 (*GATA4*), hypoxia inducible factor 1 alpha subunit (*HIF1A*), *KI67*, tumour suppressor protein 53 (*P53*), secreted phosphoprotein 1 (*SPP1*) and vascular endothelial growth factor A (*VEGFA*). The reference genes, TATA box binding protein 1 (*TBP1*) and tyrosine 3-monooxygenase/tryptophan 5-monooxygenase activation protein, zeta polypeptide (*YWHAZ*) had stable expression in testes samples as determined by geNORM V3.5 (Ghent University Hospital, Centre for Medical Genetics, Ghent, Belgium). The expression of these genes was not affected by gestational day or fetal size (*P* > 0.1). The primer sequences for all genes investigated are detailed in Table 1 [25–29]. Serial dilutions of pooled cDNA ranging from 1:5 to 1:640 in nuclease-free water were used as standards. Sample cDNA was diluted 1:25 and 2 μL of sample, standard or control were added per well. Each plate contained duplicate wells of a no template control, standards, sample cDNA and reverse transcriptase blanks. SensiFAST® SYBR Lo-ROX supermix (5 μL), 10 μmol/L forward and reverse primer stock (0.4 μL each) and water (2.2 μL). Data were analysed using qbase+ software V3.0 (Biogazelle, Ghent, Belgium). A target and run specific strategy was employed and the results, normalised to the two reference genes, were scaled to the minimum sample. The mean slope, intercept, PCR efficiency and R^2^ values are detailed in Additional file 2: Supplementary Table 1.

**Table 1:** Primer sequence details for qPCRs

Gene symbol	Gene name	Accession number	Primer sequence (5′➔ 3′)		Amplicon size, bp	Reference
*BAX*	BCL2 associated X	XM_003127290.5	Fwd	CCGAAATGTTTGCTGACG	154	[25]
			Rev	AGCCGATCTCGAAGGAAGT		
*BCL2*	B-cell lymphoma 2	XM_021099593.1	Fwd	GATAACGGAGGCTGGGATGC	147	[26]
			Rev	CTTATGGCCCAGATAGGCACC		
*CD31*	Platelet and Endothelial Cell Adhesion Molecule 1	NM_213907.1	Fwd	CCGAGGTCTGGGAACAAAGG	98	[26]
			Rev	AGCCTTCCGTTCTAGAATATCTGTT		
*DMRT1*	Doublesex and mab-3 related transcription factor 1	NM_214111.1	Fwd	AGTGTATTGTCGCCACCCAG	92	[26]
			Rev	AGCACTCCCTTTGTGCTCTC		
*GATA4*	GATA-binding protein 4	NM_214293.1	Fwd	CGACACCCTAATCTCGATATGTT	156	[26]
			Rev	CCGGCTGATGCCATTCATCT		
*HIF1A*	Hypoxia Inducible Factor 1 Alpha Subunit	NM_001123124	Fwd	CCATGCCCCAGATTCAAGAT	64	[27]
			Rev	GGTGAACTCTGTCTAGTGCTTCCA		
*KI67*	Ki67	NM_001101827.1	Fwd	AGTCTGTAAGGAAAGCCACCC	119	[26]
			Rev	ACAAAGCCCAAGCAGACAGG		
*P53*	Tumour protein p53	NM_213824.3	Fwd	GCCACTGGATGGCGAGTATT	84	[26]
			Rev	TCCAAGGCGTCATTCAGCTC		
*SPP1*	Secreted Phosphoprotein 1	X16575	Fwd	TTGGACAGCCAAGAGAAGGACAGT	121	[28]
			Rev	GCTCATTGCTCCCATCATAGGTCTTG		
*TBP1*	TATA box binding protein	DQ845178	Fwd	AACAGTTCAGTAGTTATGAGCCAGA	153	[29]
			Rev	AGATGTTCTCAAACGCTTCG		
*VEGFA*	Vascular Endothelial Growth Factor A	NM_214084	Fwd	GCCCACTGAGGAGTTCAACATC	59	[27]
			Rev	GGCCTTGGTGAGGTTTGATC		
*WYHAZ*	Tyrosine 3-monooxygenase/tryptophan 5-monooxygenase activation protein, zeta polypeptide	DQ845179	Fwd	TGATGATAAGAAAGGGATTGTGG	203	[29]
			Rev	GTTCAGCAATGGCTTCATCA		

### Statistical analysis

All statistical analyses were performed using GenStat 13.1 (VSN International Ltd., Hemel Hempstead, Hertfordshire, U.K.). Mean values were calculated for each individual sample for each parameter investigated and the normality of the distribution of the data was assessed by an Anderson-Darling test. If a *P* value of ≤ 0.05 was obtained, then the data were not considered to have a normal distribution. Log10 and square root transformations were carried out to achieve normality of the distribution of the data where required. Outliers identified by a ROUT outlier test were excluded.

Where data had a normal distribution, one and two-way ANOVA for GD and fetal size was performed, with a block for gilt to account for the common maternal environment within litter. A post-hoc Tukey test was then performed. Where data did not have a normal distribution, Kruskal-Wallis and Mann Whitney tests were performed. In all cases results were considered significant when *P* ≤ 0.05, and trending towards significant when 0.05 < *P* < 0.1.

## Results

### Temporal changes in fetal and testes weight, the number of seminiferous tubules, seminiferous tubule diameter, and number of germ cells per seminiferous tubule

As anticipated, fetal and testes weights increased with advancing gestational day (Table 2; *P* < 0.001). The mean number of tubules per 0.1 mm^2^ was decreased at GD90 compared to GD60 (Table 2; *P* < 0.001), which was unsurprising given that tubule diameter tended to be larger at GD90 compared to GD60 (Table 2; *P* = 0.09). Further, there were more germ cells per tubule at GD90 compared to GD60 (Table 2, *P* < 0.05). Seminiferous tubule area and the number of Sertoli cells per tubule was not significantly affected by GD (Table 2).

**Table 2:** Fetal testes weights and histological analyses

Variable	Gestational day			***P*** value
	45	60	90	
Average litter size	16.50 ± 1.456	16.33 ± 0.954	15.00 ± 1.304	> 0.1
Mean fetal weight, g	19.58 ± 1.211^a^	105.9 ± 6.037^b^	632.2 ± 41.28^c^	< 0.001
Mean testes weight, g		0.039 ± 0.003^a^	0.130 ± 0.020^b^	< 0.001
Mean testes weight as a percentage of fetal weight, %		0.039 ± 0.003^a^	0.024 ± 0.002^b^	< 0.001
Mean number of tubules per 0.1mm^2^		17.44 ± 0.650^a^	12.72 ± 0.837^b^	< 0.001
Mean tubule diameter, μm		51.79 ± 0.813	55.32 ± 1.476	0.09
Mean tubule area, μm^2^		2751 ± 97.56	2993 ± 149.2	> 0.10
Mean number of germ cells/tubule		3.360 ± 0.235^a^	4.671 ± 0.388^b^	< 0.05
Mean number of sertoli cells/tubule		21.33 ± 0.833	26.24 ± 3.016	> 0.1

Mean values presented ± standard error of the mean

### Fetal size was associated with altered tubule diameter at GD90

**Fig. 1:**
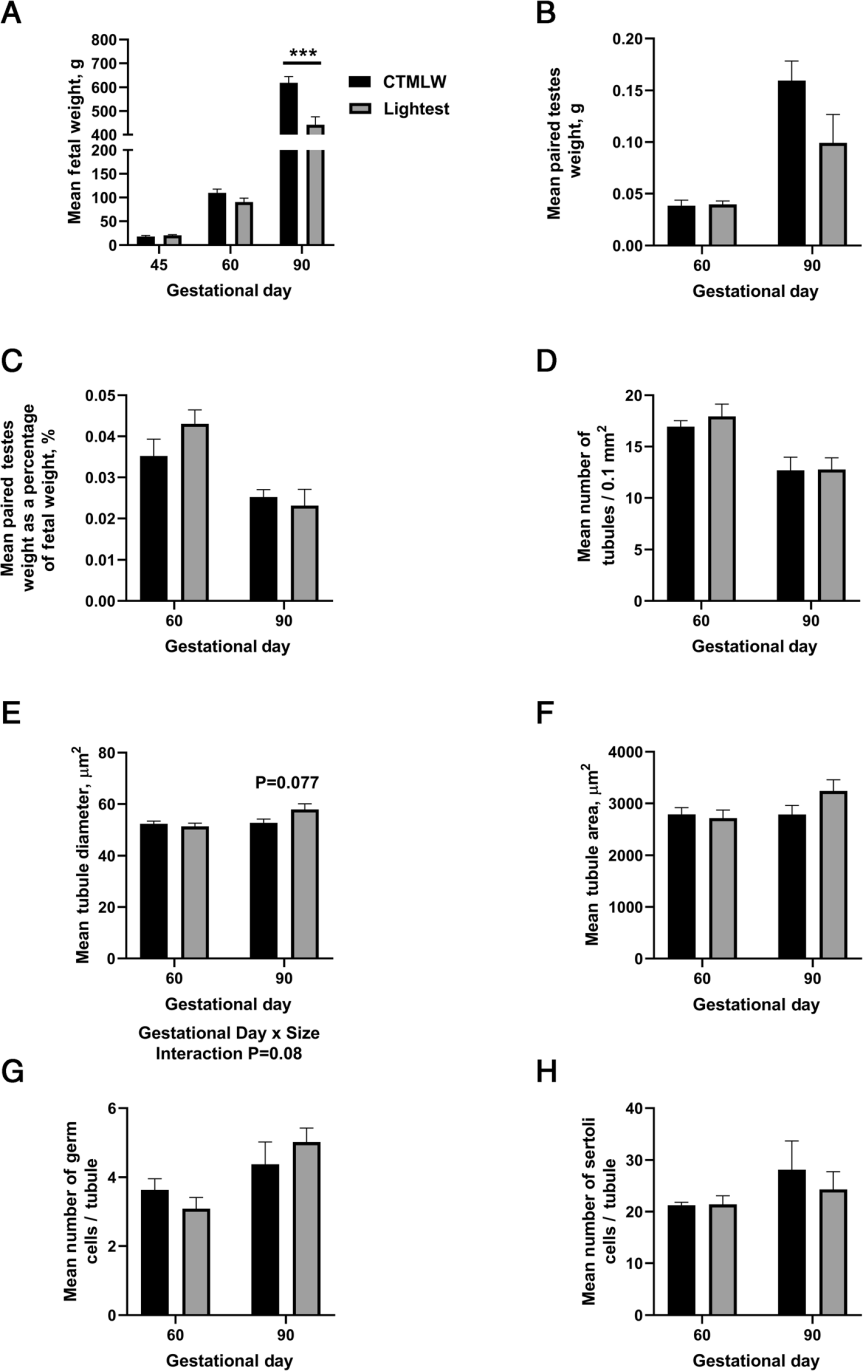
Histological analyses of fetal testes at GD60 and GD90. Fetal weights at gestational day (GD) 45, 60, and 90 (**A**), paired fetal testes weight at GD60 and 90 (**B**), and paired testes weight as a percentage of body weight at GD60 and 90 (**C**) were compared between fetuses of different size. The number of seminiferous tubules (**D**), seminiferous tubule diameter (**E**), seminiferous tubule area (**F**), number of germ cells per tubule (**G**), and number of sertoli cells per tubule (**H**) were compared between fetuses of different sizes at GD60 and 90. Mean values presented. Error bars represent S.E.M. CTMLW = closest to mean litter weight. *n* = 4–6 per fetal size per gestational day.

The lightest fetuses were significantly lighter than the CTMLW fetuses at GD90 (Fig. 1A; *P* < 0.001). Interestingly, despite the significant decrease in fetal weight in the lightest fetuses compared to the CTMLW fetuses, neither testes weight (Fig. 1B) or testes weight as a percentage of fetal weight (Fig. 1C) were significantly associated with fetal size at GD60 or GD90. The number of tubules per 0.1 mm^2^ (Fig. 1D), tubule area (Fig. 1F), and number of germ cells (Fig. 1G) and Sertoli cells (Fig. 1H) per tubule were comparable between the lightest and the CTMLW fetuses at GD60 and 90. There was a gestational day × fetal size interaction for tubule diameter, with the lightest fetuses tending to have a wider tubule diameter compared to the CTMLW fetuses at GD90 (Fig. 1E; *P* = 0.077). At GD90, fetal plasma testosterone concentration was not associated with fetal size (CTMLW 0.335 ± 0.102 ng/mL. Lightest 0.299 ± 0.096 ng/mL. *P* > 0.1).

### Temporal changes in testicular expression of *KI67* and *SPP1* mRNAs

There were temporal changes in expression of several of the candidate mRNAs investigated (Table 3). The expression of *KI67* mRNA decreased with advancing gestation (*P* < 0.01), with a statistically significant decrease at GD90 when compared with GD45. Similarly, there was a trend towards a significantly lower *BAX*: *BCL2* ratio with advancing gestation (*P* = 0.058). Testicular *SPP1* mRNA expression peaked at GD60 (*P* < 0.05) when compared with GD45 and 90. There were no significant temporal changes in the testicular expression of *BAX*, *BCL2*, *CD31*, *DMRT1*, *GATA4*, *HIF1A*, *P53,* or *VEGFA* mRNAs (Table 3).

**Table 3:** Candidate testicular mRNA expression at days 45, 60 and 90 of gestation

mRNA	Gestational day			***P*** value
	45	60	90	
*BAX*	268.7 ± 49.72	288.6 ± 46.87	230.3 ± 44.94	> 0.10
*BCL2*	2.518 ± 0.154	3.595 ± 0.277	3.994 ± 0.641	> 0.10
*BAX:BCL2* Ratio	109.8 ± 17.68	84.80 ± 12.05	55.74 ± 13.91	0.058
*CD31*	2.651 ± 0.178	3.913 ± 0.524	3.628 ± 0.407	> 0.10
*DMRT1*	3.173 ± 0.940	3.188 ± 0.489	6.234 ± 2.175	> 0.10
*GATA4*	3.155 ± 0.520	4.367 ± 0.591	2.695 ± 0.470	> 0.10
*HIF1A*	4.639 ± 0.404	4.456 ± 0.315	3.899 ± 0.402	> 0.10
*KI67*	4.381 ± 0.239^a^	3.582 ± 0.260^ab^	2.971 ± 0.300^b^	< 0.01
*P53*	2.255 ± 0.138	2.520 ± 0.188	2.179 ± 0.256	> 0.10
*SPP1*	4.418 ± 0.400^a^	9.918 ± 2.983^a^	3.116 ± 0.698^b^	< 0.05
*VEGFA*	2.062 ± 0.227	1.940 ± 0.124	2.203 ± 0.226	> 0.10

Error bars represent S.E.M. Different letters indicate that group means differ from one another within row (*P* < 0.05)

### Testes from the lightest fetuses had lower expression of *DMRT1* and *SPP1* mRNAs compared to the CTMLW fetuses at GD90

**Fig. 2:**
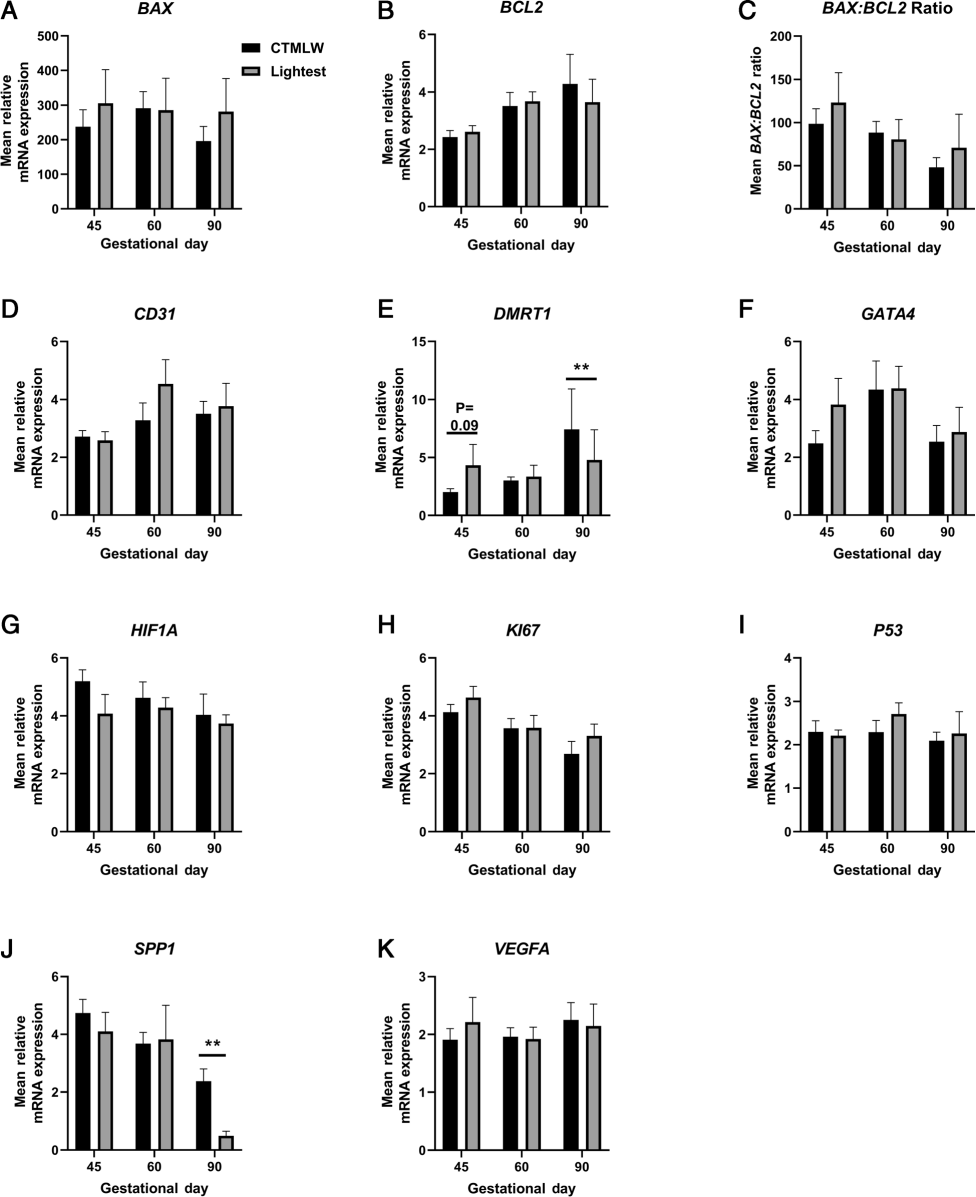
Candidate testicular mRNA expression in fetuses of different sizes at days 45, 60 and 90 of pregnancy. mRNA expression of *BAX* (**A**), *BCL2* (**B**), *BAX: BCL2* Ratio (**C**), *CD31* (**D**), *DMRT1* (**E**), *GATA4* (**F**), *HIF1A* (**G**), *KI67* (**H**), *P53* (**I**), *SPP1* (**J**) and *VEGFA* (**K**) in the lightest and closest to mean litter weight (CTMLW) porcine fetal testes at gestational days 45, 60 and 90. Error bars represent S.E.M. **P < 0.01 *n* = 5–6 per fetal size per gestational day

The mRNA expression of the candidate genes was quantified and compared between testes from the lightest and the CTMLW fetuses within GD (Fig. 2). At GD45, there was a trend towards increased expression of *DMRT1* mRNA (*P* = 0.09; Fig. 2E) in testes from the lightest fetuses compared to the CTMLW fetuses. Testes from the lightest fetuses had lower expression of *DMRT1* (*P* < 0.01; Fig. 2E), and *SPP1* (*P* < 0.01; Fig. 2J) mRNAs compared to the CTMLW fetuses at GD90. There were no statistically significant associations between fetal size and expression of *BAX*, *BCL2*, *BAX: BCL2* Ratio, *CD31*, *GATA4*, *HIF1A*, *KI67*, *P53*, or *VEGFA* mRNAs (Fig. 2).

## Discussion

Improved understanding of the mechanisms and timing of developmental changes in the fetal testes is essential to improve knowledge of how male pigs reach their reproductive potential postnatally. Whilst Pontelo et al. [20] recently described temporal changes in testicular size and structure during porcine fetal development, the expression profiles of genes associated with testicular development and function remains poorly understood. In this study, the temporal expression profile of selected mRNAs with central roles in apoptosis (*BAX* and *BCL2*), proliferation (*KI67*), angiogenesis (*CD31*, *HIF1A* and *VEGFA*), and the testicular extracellular matrix (*SPP1*) and function (*DMRT1* and *GATA4)* was determined.

Striking changes in the structure of the testes occur during gestation to prepare the testes for its important and essential role in male fertility in postnatal life. SPP1, also known as osteopontin, is an extra-cellular matrix (ECM) protein which functions by binding to integrin receptors expressed on the cell surface to promote cellular adhesion and communication [30]. Given the important role of this extracellular matrix protein in other tissues [31–33], the observed gestational stage-related changes in *SPP1* mRNA expression are perhaps unsurprising. SPP1 protein is localised to the germ cells within seminiferous tubules, supporting cells in the seminiferous tubules and the interstitial Leydig cells of boar testes [34]. Given the striking change in *SPP1* mRNA expression observed during late gestation, further studies should be performed to characterise the role of SPP1, integrins, and other ligands of the integrin receptors such as fibronectin, in the fetal testes, to gain better understanding of the mechanisms involved in testicular development.

In this study, neither testes weight or testes weight as a percentage of fetal weight were associated with fetal size at GD60 or GD90, suggesting that gonadal growth may be spared in these growth restricted fetuses, consistent with our previous findings in female fetuses at the same stages of gestation [26]. Similar findings have been reported in boars at 8 days and 8 months postnatally, suggesting that whilst overall testicular weight may be reduced in IUGR relative to normal weight boars, testicular weight is proportional to body size throughout development [7]. Whilst it is known that reproductive function is impaired postnatally in boars that are lighter at birth, and this study has demonstrated that there are alterations in testicular mRNA expression during fetal life, the idea that relative testicular growth is proportional to fetal size interesting and warrants further investigation.

IUGR boars postnatally have impaired testicular development, with decreased numbers of germ cells, testicular volume, semen volume, and fewer spermatozoa per ejaculate postnatally when compared to normally-grown piglets [8, 9]. Neonatal testes from the lightest boars have fewer total Sertoli, Leydig, and germ cells compared to the heaviest boars [7, 8]. Yet, at 10 months of age there was no association between seminiferous tubule diameter and birth weight [8]. At 8–10 months of age, there were fewer pachytene spermatocytes, round spermatids, elongated spermatids, and lower semen volume and number of spermatozoa per ejaculate in the lightest boars compared to normal or heavy birthweight boars [7, 9]. Whilst Smit et al. [8] reported lower total numbers of germ, Sertoli and Leydig cells, they did note that the number of each of these cell types per gram of testis weight was comparable. The developmental trajectory of testicular development in the lightest fetuses compared to their normally grown littermates remains poorly understood. In the present study, no association between fetal size and the number of tubules per 0.1 mm^2^, tubule area, germ cell number per tubule, or Sertoli cell number per tubule were observed at GD60 or 90. Pontelo et al. [20] reported a significant correlation between fetal weight and Sertoli cell number in testes from days 50, 80 and 106 of gestation. However, these correlations appear to have been performed on samples combined from multiple gestational days (GD50, 80 and 106), so they are perhaps not directly comparable with the data in the present study, which presumably more accurately reflect the relationships between fetal size and testicular development within gestational day. It has been postulated that testicular cell proliferation is greatest during the final trimester of pregnancy in the pig [20]. Given this, it could be hypothesised that the reproductive phenotype of IUGR boars postnatally originates due to alterations in proliferation and/or apoptosis during the final stages of fetal development, perhaps between day 90 and parturition.

Despite the appreciation that testosterone is vital for both the regulation of fetal development and postnatal reproduction, the relationship between plasma testosterone and boar size both prenatally and postnatally remains under investigated. In the current study, no association between fetal size and plasma testosterone concentration was observed at GD90. This is consistent with data from neonatal boars where plasma testosterone and the mRNA expression of 17α-hydroxylase were not associated with birth weight at 8 days and 8 months postnatally [7]. Similarly, Lin et al. [9] suggested that there was no association between circulating testosterone concentrations and birth weight in boars at postnatal days 82 and 242.

Given the striking differences that have been reported in the literature regarding variation in porcine fetal size from an early stage of gestation [14–18], and the significant development of the testes during early and mid-gestation, we hypothesized that significant differences in the testicular expression of candidate mRNAs involved in extracellular-matrix remodelling, proliferation and apoptosis would be observed across gestational stages and between littermates of different weight. Given the postnatal phenotype of IUGR boars, it could be speculated that enhanced apoptosis and impaired cellular proliferation would be observed in the testes of the lightest compared to the CTMLW fetuses. In this study, no association between fetal size and the expression of mRNAs involved in apoptosis and proliferation were observed at any stage investigated. However, it has been suggested that testicular cellular proliferation is greatest during the final trimester of pregnancy in the pig [20]. Given this, it could be hypothesised that the reproductive phenotype of IUGR boars observed postnatally originates due to alterations in cellular apoptosis and proliferation either during the initial stages of testes development or during the final stages of fetal development.

As described earlier, the ECM protein SPP1 is important for regulation of cellular adhesion and communication, and is known to be expressed in new-born boar testes [34]. In the present study, the lightest fetuses had lower testicular expression of *SPP1* mRNA than the CTMLW fetuses at GD90. Considering the localisation of this protein in the testes to the germ cells within seminiferous tubules, supporting cells in the seminiferous tubules and the interstitial Leydig cells of new-born boar testes [34], and its vital role in the regulation of ECM structure, further assessment of the role of integrins and their ligands may provide interesting insights into the mechanisms regulating the impaired reproductive phenotype observed in IUGR boars postnatally.

Sex determination occurs early in embryonic development in mammalian species, whereby the bipotential gonad differentiates into either a testis or an ovary depending upon the sex chromosomes present in the embryo [35]. In the male embryo, the Y-linked gene *SRY* is critical for the development of the testes, with *SRY* expression in pre-Sertoli cells inducing upregulation of *SOX9*, which then induces the expression of many testes determining genes, including *DMRT1*. *DMRT1* is a highly conserved gene which is expressed in greater abundance in the testis compared to the ovary in the embryo [36, 37]. At GD90, the expression of *DMRT1* mRNA in testes was lower in the lightest compared to the CTMLW fetuses. Whilst it is well-established that DMRT1 has an important role in early testes development, it is also essential for the maintenance of Sertoli cells, both in fetal and postnatal life, and thus ultimately it is important for the maintenance of testicular function [38]. The finding of decreased *DMRT1* mRNA expression at GD90 in the testes of the lightest fetuses compared to the CTMLW fetuses warrants further investigation. Reduced expression of *DMRT1* by the testes from the lightest fetuses may be indicative of less maintenance of the Sertoli cell population, which may in turn lead to decreased Sertoli cell number in postnatal life. As Sertoli cells play an essential role in the regulation of spermatogenesis, and given the reported decrease in spermatocytes, round spermatids, elongated spermatids, and lower semen volume and number of spermatozoa per ejaculate in IUGR boars [7, 9], reduced DMRT1 signalling could in this way lead to the impaired reproductive phenotype observed postnatally in these animals. In IUGR rats, Taqman arrays have revealed alterations in genes associated with Sertoli cell and Leydig cell function during early postnatal life [39]. Further, RNASeq experiments comparing testicular RNA expression between Meishan and Duroc boars at postnatal days 20, 75, and 270 identified many genes associated with attainment of puberty and spermatogenesis [40]. Further investigation of some of the candidate genes validated in these studies could provide interesting insights into the mechanisms governing altered postnatal reproductive performance in IUGR boars.

## Conclusions

This study provides novel insights into the temporal histological and transcriptomic changes that occur during normal fetal testicular development. Based on morphological and gene expression analyses, our results suggest that the developmental trajectory of IUGR testes does not deviate from that of normally sized fetuses until late in gestation. This study suggests that the expression of *SPP1* and *DMRT1* mRNAs is lower in testes from the lightest fetuses at GD90, providing a platform for further mechanistic investigations. Given the key role of SPP1 in the regulation of the extracellular matrix, and of DMRT1 in sex determination and the maintenance of Sertoli cells, further studies are warranted to elucidate their involvement in the impaired reproductive potential of IUGR boars. Improving the understanding of the mechanisms regulating fetal testes development is critical to improve the understanding of the regulation of postnatal boar fertility and is vital to improve the success of the pig industry.

## Supplementary Information

**Additional file 1.** Supplementary Fig. 1: Representative Images of GATA4 Immunohistochemistry in Fetal Testes. Immunohistochemistry revealed that GATA4 protein is expressed by the somatic cells in both the lightest (B and D) and closest to mean litter weight (CTMLW) (A and C) at both gestational day (GD) 60 (A and B) and 90 (C and D). Rabbit IgG controls at an equivalent protein concentration were utilised as a negative control (E). Scale bars represent 100 μm.

**Additional file 2.** Supplementary Table 1: Quantitative polymerase chain reaction calibration curve data.

## Data Availability

The authors confirm that the data supporting the findings of this study are available within the article and its supplementary materials.

## Abbreviations

CTMLW: Closest to mean litter weight
ECM: Extracellular matrix
ELISA: Enzyme-linked immunoassay
GD: Gestational day
IUGR: Intrauterine growth restricted
PBS: Phosphate buffered saline
PCR: Polymerase chain reaction
qPCR: Quantitative polymerase chain reaction
RINe: RNA integrity number equivalent
SEM: Standard error of the mean
