# Supplementary figures and images for: Associations between testicular development and fetal size in the pig
Source: J Anim Sci Biotechnol. 2022 Mar 15;13:24. doi: 10.1186/s40104-022-00678-3 (PMC8922848; doi:10.1186/s40104-022-00678-3)

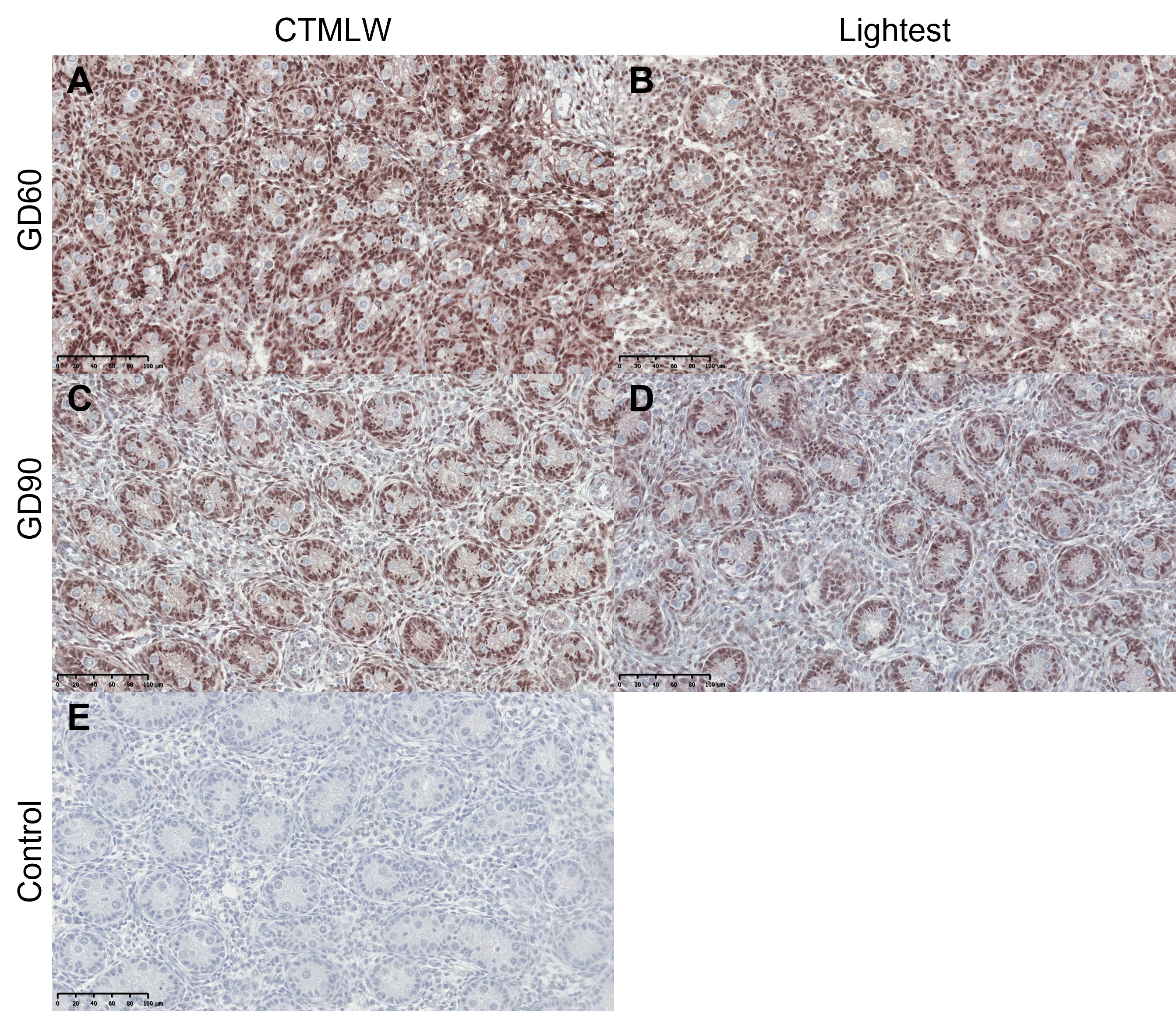

Supplement: Supplementary file 1 — Additional file 1. Supplementary Fig. 1: Representative Images of GATA4 Immunohistochemistry in Fetal Testes. Immunohistochemistry revealed that GATA4 protein is expressed by the somatic cells in both the lightest (B and D) and closest to mean litter weight (CTMLW) (A and C) at both gestational day (GD) 60 (A and B) and 90 (C and D). Rabbit IgG controls at an equivalent protein concentration were utilised as a negative control (E). Scale bars represent 100 μm. [file 40104_2022_678_MOESM1_ESM.tif]
