# Supplementary material for: Associations between testicular development and fetal size in the pig
Source: J Anim Sci Biotechnol. 2022 Mar 15;13:24. doi: 10.1186/s40104-022-00678-3 (PMC8922848; doi:10.1186/s40104-022-00678-3)
Supplement: Supplementary file 2 — Additional file 2. Supplementary Table 1: Quantitative polymerase chain reaction calibration curve data. [file 40104_2022_678_MOESM2_ESM.docx]

Supplementary Table 1: Quantitative polymerase chain reaction calibration curve data.

| **Gene** | **Slope** | **Intercept** | **Amplification Efficiency, %** | **RSq** |
| --- | --- | --- | --- | --- |
| *BAX* | -3.392 | 29.287 | 97.2 | 0.940 |
| *BCL2* | -3.402 | 28.903 | 96.8 | 0.940 |
| *CD31* | -3.538 | 23.306 | 91.7 | 0.985 |
| *DMRT1* | -3.430 | 26.177 | 95.7 | 0.993 |
| *GATA4* | -3.235 | 26.834 | 103.8 | 0.992 |
| *HIF1A* | -3.429 | 26.201 | 95.7 | 0.994 |
| *KI67* | -3.239 | 26.868 | 103.6 | 0.992 |
| *P53* | -3.450 | 28.035 | 94.9 | 0.964 |
| *PTGFR* | -3.573 | 25.987 | 90.5 | 0.979 |
| *SPP1* | -3.531 | 23.651 | 91.9 | 0.951 |
| *TBP* | -3.439 | 22.978 | 95.3 | 0.992 |
| *VEGFA* | -3.233 | 24.670 | 103.9 | 0.993 |
| *YWHAZ* | -3.471 | 20.319 | 94.2 | 0.989 |
